# 

**Published:** 1898-04-23

**Authors:** 


**Notices of Births, Deaths, and Marriages** are inserted in this column at a charge of 2s. 6d. for an announcement not exceeding four lines.

### NOTICE TO CORRESPONDENTS.

All MS., Letters, Books for Review, and other matters intended for the Editor should be addressed The Editor, "The Hospital," The Hospital Building, 28 & 29, Southampton Street, Strand, W.C.

The Editor cannot undertake to return rejected MS., even when accompanied by stamped directed envelope.

**NOTICE.—Vol. XXIII. of "The Hospital" (October, 1897, to March, 1898), in handsome cloth binding, will shortly be on sale at the Office of this Journal, price 7s. 6d. Cases for binding the Numbers contained in this Volume, 1s. 6d. Index to Vol. XXIII., 2½d., post free.**

**The Monthly Part for May will be ready on May 2nd. Price 6d., by post 8d.**

**Notice to Advertisers and Subscribers.**

All Advertisements, Orders for Copies of The Hospital, and Business Communications should be addressed to **THE MANAGER, (Not to The Editor),** The Hospital, The Hospital Building, 28 & 29, Southampton Street, Strand, London, W.C.

The Cost of Subscription, post free, is as follows:—Per week, 2½d. per quarter, 2s. 8d.; per half-year, 5s. 3d.; per annum, 10s. 6d. Foreign:—Per annum, 15s. 2d.; payable, in all cases, in advance.

### Scraps and Gleanings.

Baroness Hirsch has sent the sum of £150 as a donation to the North-Eastern Hospital for Children.

A well-known firm of piano makers has just brought out an "Invalid Piano," whose softened chords it is claimed will not jar on irritable nerves.

The Duke and Duchess of Fife have promised to open a grand bazaar on May 4th, at the Royal Palace Hotel, Kensington, in aid of the Society for Providing Homes for Waifs and Strays.

A suggestion has been seriously made by an important New York insurance company that a palmist should be attached to their office, whose business it would be to calculate the probable life-term of would-be policy-holders!

The Foleshill Fever Hospital has just been lighted by oil conveyed to the burners through pipes in a similar manner to gas from a tank outside the building, the Imperial Oil Company, of Coventry, being the contractors. This is of interest, as being the first public building so lighted, but others are shortly to be dealt with in the same manner.

Visitors to Dublin are immediately struck by the life saving provision made along the line of the River Liffey. On every lamp-post a life-belt hangs suspended, so that if a Dublin man or woman finds his way into the river, either by accident or design, a very ready means of saving him from drowning is in the hands of the police or passers-by. Apparently this is a novelty in civic precautions, for one does not remember to have seen it elsewhere.

### The Student of Medicine.

### APPOINTMENTS.

J. W. Alexander, M.R.C.P., L.R.C.S.Edin., appointed Medical Officer for the Armley District and the Workhouse of the Bramley Union. P. Bayley, L.R.C.P., L.R.C.S., L.M.Edin., appointed Second Assistant Medical Officer of the City of London Asylum, near Dartford. T. W. W. Bovey, M.R.C.S.Eng., L.R.C.P.Lond., appointed House Physician to the Norfolk and Norwich Hospital, Norwich. J. C. Bowie, M.B., C.M.Aberd., appointed Medical Officer and Public Vaccinator to the Parishes of Sandsting and Aithsting, Shetland. H. Burton, L.R.C.P.I., M.R.C.S.Eng., appointed Medical Officer of Health to the Marple Urban District Council. C. Dixson, M.D.Dunelm, M.R.C.S.Eng., appointed Honorary Anæsthetist to Brentford Cottage Hospital and Dispensary. E. Du Cane, B.A., M.B., B.Ch., appointed Resident Medical Officer, Christ College, Brecon. W. Fairbanks, M.D.Edin., appointed Medical Officer of the Workhouse of the Wells Union. A. D. Forbes, M.B.Aberd., appointed Medical Officer for the No. 7 (Horsmonden) District of the Tonbridge Union. J. R. Hatfield, L.R.C.P., L.R.C.S.Edin., L.F.P.S., Glasg., appointed Medical Officer for the Worthen District of the Forder Union. J. E. Hickman, M.R.C.S., L.R.C.P.Lond., appointed Medical Officer for the Coleford District of the Frome Union. F. W. Mason, L.R.C.P., L.R.C.S., appointed Medical Officer for the No. 4 District of the Flaxton Union. S. G. Morris, M.D., appointed Medical Officer for the Western District of the Llandilofawr Union. A. S. Robinson, B.A., M.B., B.C.Cantab., L.S.A.Lond., appointed Medical Officer and Public Vaccinator to the Kirkleatham District of the Guisborough Union. F. G. Scott, M.R.C.S., L.R.C.P.Lond., appointed Medical Officer for the Gillingham District of the Medway Union. J. W. Smith, M.D., appointed Medical Officer of Health to the Ryton Urban District Council. A. F. Stabb, M.B., B.C.Cantab., M.R.C.P., appointed Assistant Obstetric Physician to St. George's Hospital. J. P. S. Ward, M.R.C.S., L.R.C.P., appointed Physician's Assistant to the Plymouth Public Dispensary.

